# Book Reviews

**Published:** 1820-03

**Authors:** 


### CRITICAL ANALYSIS

### OF

### RECENT PUBLICATIONS, IN THE DIFFERENT BRANCHES OF MEDICINE AND SURGERY.

"I would have men know, that, though I reprehend the easie passing over of the causes of things, "by ascribing them to secret and hidden vertues and properties; (for this hath arrested and laid "asleepe all true enquiry and indications;) yet I doe not understand but that, in the practical "part of knowledge much will be left to experience and probation, whereunto indication cannot "so fully reach: and this not only in *specie*, but in *individuo*. Yet it was well said, *Vere scire* "*esse per causas scire*."—BACON.

*A History of the High Operation for the Stone, by Incision above the Pubis, with Observations on the Advantages attending it; and an Account of the various Methods of Lithotomy, from the earliest Periods to the present Time.* By J. C. CARPUE, F.R.S. Member of the Royal College of Surgeons in London, and formerly Surgeon to the York Hospital, Chelsea. 8vo. pp. 104; with several Plates. Longman and Co. 1819.

A CRITICAL history of the several modes of cutting for the stone that have been in use, especially in relation to the advantages and disadvantages of each, the reasons for which some of them have been relinquished, and the means of obviating the objections made to them; with a series of arguments in favour of the restoration of the practice of the High Operation; constitute the subjects of the present work; and it is long since we before perused one with feelings of deeper interest; for it makes it apparent that we have selected from amongst the known methods that which, in the generality of cases, is neither the least painful when endured, the most certain in its immediate object, nor the least dangerous in its consequences. Such an assertion must, on a first consideration, appear to be ill-founded: it will not seem probable that a better method than that now in general use can have been abrogated at a period when surgery was practised by enlightened men, or have been almost universally neglected for the long interval that has elapsed from the time of BASEILHAC to the present day. This idea, it seems, has been strongly impressed on the mind of the author; and hence, probably, he was induced to undertake the arduous labour of writing the history of this part of surgery, at the same time that he advanced his more immediate arguments in favour of the measure above designated. In making the statement we have done respecting the result of the investigation of this question, we only express what is now acknowledged by many English surgeons of the first eminence; and in France, the statue of FRERE COME, who formerly was regarded only as a celebrated charlatan, has lately been placed in the hall of the College of the Faculty of Paris.

The *Celsian method*, the *cutting on the gripe*, or that by the *lesser apparatus*, as the same mode of performing lithotomy has been differently called, was universally adopted until the commencement of the sixteenth century, when JOHANNES DE ROMANIS invented the operation called, that by the *apparatus major*, or the *Sectio Mariana*, from SANCTUS MARIANUS BAROLITANUS, who published the first description of it in 1535. This method strongly indicates the circumstances under which it was adopted: want of accurate anatomical knowledge; blind submission to the ancient authorities; and a disposition to make success in the attempt to extract the stone, and the leaving the patient alive immediately after it, the chief points for consideration, rather than the final consequences of the operation. Not so with those of PIERRE FRANCO, whose name Mr. CARPUE has rescued from almost total oblivion, and placed in the list of fame amongst those of the greatest improvers of the healing art. *Nihil præter* INVIDIA *medicorum*, has been a famous proverb; and FRANCO, as well as VESALIUS, BORDEU, and many others, was a victim of its influence.

Pierre Franco was born at Turriere in Provence, about the commencement of the sixteenth century. He taught anatomy at Friburg and Lausanne, and published his first work, entitled *Petit Traité contenant une des parties principales de Chirurgie laquelle les Chirurgiens Herniaires exercent*, in 1556. It appears from this work that he was not only the inventor of the *lateral operation*, but that he also successfully practised, in one instance, that above the pubes. Franco was, in every respect, a great man. He was a good surgeon, very far superior to his contemporaries, an original and judicious reasoner, and a shrewd observer of men and manners. His method of performing the lateral operation, sufficiently indicates the first assertion. He published the history of his case of the high operation in his works; which he resorted to, he says, because the patient was *suffering extreme agony*, (he had made the lateral incision, and could not by this means extract the stone, from its great size, that of a hen's egg,) *and the parents desired that he should die rather than continue to live in such misery; and I, too, wished to avoid being reproached for not having been able to extract the stone, (which was great folly on my part,)* &c.: and, speaking of the nature of the operation indicated, he says, *yet I do not advise any one to do the like.*

Here he seems, as Mr. Carpue remarks, to have had futurity in his view; or why did he publish this case? He had erred against the axiom of Hippocrates, that wounds of the bladder are necessarily mortal; and he knew that some food must be given to envy, which he chose to supply from his moral character rather than in the operation itself, which he thought might at some future time be appreciated, and resorted to with advantage. Hence his expressive and appropriate motto, *Il faut endurer pour durer*.

The instruments he used in the lateral operation were, a staff with a groove, a clasped scalpel called a razor, which cuts at the point on both sides, and a blunt gorget. The following is the mode of performing it.

"The staff is to be introduced into the urethra, and given to an assistant, and must be pushed towards the perinæum. An incision is to be made between the testicle and fundament; the razor is then to be introduced into the groove of the staff. This being done, you must slide the said razor into the groove, cutting the neck of the bladder upon the cavity of this. You must now take out the razor and take the gorget, and with the point find out the groove of the staff. To do this the staff must be lowered, in order that it may slide upwards into the groove. Then the gorget must be passed, following always its point in the groove, till the gorget comes out of the cleft of the groove. The gorget being then in the bladder, being well assured that it is there, you must draw out the staff, the gorget remaining in the bladder; after which you will take the forceps, and pass them in upon the gorget as far as the body of the bladder. When in, you will take the gorget out, and close the forceps when the stone is in them.

"Franco observes, that the incision must be made according to the size of the stone. Wound to be kept open."

Franco was also the inventor of another mode of proceeding in this operation, which he calls the operation *en deux temps*, which he resorted to several times with success, in cases where the stone could not in the first instance be extracted by the lateral operation, without risking the life of the patient by the long continuance of the irritation: he ceased his attempts, placed the patient in bed, used tepid applications, and waited until the irritation and consequent inflammation had subsided. Then, if the stone did not present (as it will commonly do) at the wound, he introduced his forceps, and found that he could easily extract the stone, by the assistance of a finger passed into the rectum and pressure on the abdomen. This method has been since adopted several times, and with success. It is evidently deserving of more attention than it has yet received: it is not even noticed in other histories of lithotomy.

There is no decisive evidence of the lateral operation having been performed, after Franco, until it was employed by Jacques Beaulieu, commonly called Frere Jacques, first at Besançon, and afterwards, in 1697, at Paris; though Mr. Carpue thinks it probable that the use of it had been continued in the south of France, and especially by RAOUX. It is Frère Jacques to whom Mr. John Bell, and other historians of this part of surgery, attributed the invention of the lateral operation. The next operator in this way was, apparently, RAU; but it is very curious that, as Heister states, when Rau came to that part of his course of Operative Surgery which treats of lithotomy, his phrase was "that he had nothing to say upon that head, because it was the means by which he got his living; and I had rather be silent than propose any thing that might mislead you from the truth: but, if you can learn it by seeing me perform it upon living subjects, you are welcome; and for the rest, you may read Celsus." Heister was the first after Rau who performed the lateral operation, at the hospital at Oudenarde.

Rau's success had been very considerable since the time of Frère Jacques' operation in Holland, though, previously to this period, he having practised the methods by the greater and lesser apparatus, a great proportion of his patients died. His success stimulated the surgeons of various parts of Europe, and, amongst others, Dr. BAMBER and Mr. CHESELDEN, to abandon the high operation, which, prior to that period, according to Dr. DOUGLASS, they had practised with great success.

In the year 1740, Mr. SHARP says, that Mr. SERJEANT HAWKINS seems to have fallen upon a most ingenious contrivance, by making his *gorget cut on the right side*, &c. From the time of Hawkins to the present day, the cutting gorget has been most generally used, though the form and construction of that instrument have been varied by several surgeons.

The lateral operation had not been performed in France from the time of MARESCHAL, (who adopted it from Frère Jacques, and, by means of his superior anatomical knowledge and authority, had wrested the practice of lithotomy out of the hands of the latter,) until that of MORAND, who, seeing with regret the number of deaths that arose from the use of the apparatus major, came to London to learn the practice of Cheselden; and from that time the lateral operation has been usually practised in France.

After Franco, we hear no more of the high operation, till the year 1581, when ROUSSET ventured to advise it, though he never performed it. Describing Franco's operation, he says, "I cannot forbear very much wondering why he should endeavour to discourage others from attempting the like. Did that good man envy mankind the happiness of the invention?" He made several experiments on dead bodies, to show its practicability and probable advantages.

The next step in the history of this operation, is to Dr. GREENFIELD, who, in his work entitled a *Complete Treatise on*

*the Stone and Gravel*, published in 1710, says, after having given an account of Franco's operation:

"*Una hirundo non facit ver.*

**"Yet I once had a patient in Long-lane, Moorfields, upon whom I was obliged to perform this high operation, and very successfully extracted the stone, by making an incision near the groin, the patient soon recovering; which shews that wounds in the fibrous parts of the bladder are not always mortal."**

Douglass takes great merit to himself for having resumed the use of this operation. He resorted to it but four times. One of his patients, a child but three years old, died of *convulsions* about *fifteen hours* after the operation. This, therefore, furnishes no direct argument against the preference of this method.

Cheselden performed the high operation but nine times, one of which was unsuccessful; but here death was not fairly attributable to the operation. Dissection showed such disease of the kidneys, that Dr. Coteswortth, who attended the patient, stated it as his opinion, that he had lived longer than he could have done if he had not been cut.

Mr. Pye, of Bristol, published some account of the high operation in 1724. He wrote against it; but the cases on which he founds his arguments, really furnish hardly the slightest objections to it. The patient of the first fatal case he relates, was a bad subject for any operation of the kind, and it was apparently very ill performed. The second had severe disease of the bladder of long standing, its coats being *ulcerated and very fungous;* and the neck, where the stone lodged, was *very schirrous and full of deep ulcers:* and, in the last, dissection showed that the patient died from disease of one of the kidneys; the *parts adjacent to the bladder* (the one-and-twentieth day after the operation) *were free from any inflammation, and the wound incurned with the muscles of the belly.*

Mr. Thornhill, of Bristol, performed the high operation ten times, the first in 1722; two of which cases were fatal: of the first of which, Dr. Middleton, who gives the histories of them, says, the patient, a boy fourteen years of age, "seemed to me to have a cachexy, with symptoms of an ascites, on which account I dissuaded Mr. Thornhill from the operation, who shifted it above two months, and would willingly have been excused; but he was at last prevailed on by the daily solicitations of the boy's relations, and the pressing desires of some of his own friends." From the diseased state of the bladder, (which was found on dissection to be *very schirrous, and in some places above an inch thick,*) the stone being also large, (four inches long, and larger at one end than the other,) there was great difficulty in extracting it. He died on the day after the operation.

"His body was opened: both his kidneys were very large and flabby. There were two ureters came from the right kidney, which were of a monstrous size; and half-way down to the bladder they united into one, which was five times as large as the natural. From the left kidney the ureter came off monstrously large, in which was contained above a quart of urine. The bladder was very schirrous, and in some places above an inch thick. The peritonæum was gangrened; the rest of the viscera were all sound."

In the other fatal case, the peritonæum was wounded, and the intestines came down between the hands of the operator. This accident is certainly a result of the operation, but it is one which is not likely to happen when it is practised after the manner of Frère Côme, which will be presently described. The wound was dressed in a very injudicious manner in this case; indeed, in a way expressly calculated to favour effusion of urine. Great inflammation came on, with convulsions on the third day, when the boy died. Bad treatment must here share the blame.

The ten cases above designated are narrated at length by Dr. Middleton; and he concludes by adding, that "there have been five or six more cut by Mr. Thornhill; of these, one died the third day after the operation: when he was opened, we observed *a schirrous tumor in his bladder*."

Mr. Macgill, of Edinburgh, had been performing the high operation about the same time. In a letter to Dr. Douglass, dated October 1723, he mentions having done it in four cases, all of which terminated happily; though in three of them there existed circumstances unfavourable to the success of any operation of the kind.

Heister performed it twice, and with success in both instances; though he resorted to it only because he could not in those cases have extracted the stone through the peritonæum. Yet he points out a circumstance in the operation which, as conformed to by Frère Côme, is a great improvement in it: the effecting it without previous injection of the bladder; a part of it often complained of by the patients to whom we have above alluded, and which probably not unfrequently contributed to the danger of the operation.

Morand was obliged to choose this method in one instance, in 1727, by the peremptory desire of an invalid old soldier. The patient died on the forty-third day after it, but indubitably from a combination of adventitious circumstances,—as, the shattered state of his constitution, his imprudencies with respect to diet, and in the manner of using a catheter.

He afterwards resorted to it by voluntary choice; and states that he could give the histories of forty cases, out of which thirty-five recovered. No one has better explained the cause of the general neglect of it, than he has done, in the remark which terminates his observations, that " the influence fashion has over the most essential things, is no less inconceivable than true."

From the period here alluded to, the high operation was not performed in France, until it was practised, about the middle of the last century, by John Baseilhac, better known by the name of Frère Côme, whom Mr. Carpue shows to have been a man well educated in surgery; not, as a modern writer on the subject says, " a priest, ignorant, like Frère Jacques, of anatomy, or the principles of our science." He first, however, performed the lateral operation with his *bistoire cachée*, but he afterwards used the high operation in many cases; though he remarks, that it will require an experienced lithotomist to decide in what cases this operation is necessary. In females, he says, speaking generally, the high operation is far superior to any other. But he soon found that the injection of the bladder was not only an evil in itself, but also that it frequently could not be effected so as to answer the purpose for which it was intended,—the raising it above the bones of the pubes. He was therefore induced to make a number of experiments on the dead body; the chief results of which were, the invention of the *sonde-de-dard*, the incision in the peritonæum; and, in a word, the mode of effecting the operation which Mr. Carpue desires to bring again into general use. Besides the sonde-de-dard, a *trois-quarts bistouri* (a trochar containing a bistoury), and a *suspensor of the bladder*, should be mentioned amongst the instruments he invented of great importance, in regard to the success of the operation. The latter is to be inserted into the bladder after the incision has been made in it, in order that the operator may have both his hands at liberty for the extraction of the stone. The former will prevent the hazard of wounding the peritonæum. We shall give Mr. Carpue's directions for the whole course of this operation, and the subsequent management, in his own words.

" A staff is introduced into the bladder. An incision is made through the integuments of the peritonæum, and a small incision into the membranous part of the urethra; a director is introduced into the bladder, upon the staff: the staff is withdrawn; the sonde-de-dard is introduced upon the director into the bladder: the director is now withdrawn; the sonde-de-dard is held by an assistant. An incision is then made, three or four inches in length, through the integuments of the abdomen. The trocar-bistouri is passed through the linea alba, close to the posterior part of the pubis. The concealed blade is opened, by means of which the lower part of the linea alba is divided. A probe-pointed bistouri is introduced, through the opening which had been made by the concealed bistouri, into the lower part of the linea alba, and the incision is continued by means of this instrument. The operator takes the sonde-de-dard from the assistant with his right hand, and pushes it forward, by which means he elevates the bladder above the pubis. The assistant now holds the sonde-de-dard, and the surgeon with his right hand pushes the stilet (which is contained in the cannula of the sonde-de-dard) through the superior and anterior part of the bladder; he takes hold of the end of the stilet with his left hand, and passes a probe-pointed bistouri along the groove (which is in the anterior part of the stilet), and makes an incision in the superior anterior part of the bladder. He passes the index-finger of his left hand into the bladder, by means of which he supports it. The stilet is withdrawn from the cannula of the sonde-de-dard, which is now lowered and held by an assistant; the operator introduces the suspensor of the bladder, which is held by an assistant. The stone is now to be withdrawn with the finger and thumb, which, if small, is done with great ease. If the bladder is large, a finger is introduced per rectum, by which the bladder is elevated, and the stone more readily found. If the stone should be in an excavation, and the bladder is not of a very large size, it may be discovered with the finger, by means of which the surgeon will know whether a scoop, or what kind of forceps, is indicated. If the stone should be of such magnitude as is represented in the case of the widow Donneri, Frère Côme's forceps must be used: if the stone should adhere to the bladder, or be contained in a cist, the means used by M. Baseilhac and Sir Everard Home should be resorted to. When the stone has been extracted, Dr. Souberbielle introduces a silver wire through the cannula of the sonde-de-dard, and passes it through the wound made in the linea alba; this is held while the sonde-de-dard is withdrawn; a flexible gum catheter is now passed into the bladder, through the wound in the membranous part of the urethra, by means of this wire. The wire is now withdrawn. The catheter is confined in this situation, by means of tapes passed round the thighs and pelvis of the patient; a bladder is tied to it, to receive the urine. Though this method of introducing the catheter is done in a very short time, yet I think that, if the point of the catheter were introduced into the sonde-de dard, the sonde-de-dard might be brought through the opening in the linea alba, and by this means the catheter might be conducted into the bladder with the greatest facility; or the cannula of the sonde-de-dard might be made of flexible gum, which, in place of silver, would contain the stilet, and this might remain in the bladder, and the lower part might be cut off, thus answering every purpose.

"*Method of dressing the patient.*—A piece of soft linen, half an inch wide and six or eight inches long, is to be introduced, by means of a pair of forceps, into the bottom of the bladder; the edges of the wound are to be covered with lint. To prevent the urine excoriating the parts, the linen is to be allowed to pass over the pubis on either side, and by this means the portion of the urine which is not carried away by the catheter, will be carried off by means of this linen.*

* If you place a piece of linen in the bottom of a cup filled with water, and let the ends rest upon a plate, in three or four hours the whole of the water will be in the plate.

"Lint and light dressings are to be applied to the wound, and a bandage passed round the abdomen.

"Great care is to be taken that the catheter is kept open; a stilet should occasionally be passed. Much attention should be paid to the subsequent dressings. Usually, on the third day, the linen may be taken from the bladder, as by that time the greatest part of the urine will pass by the catheter. By this time the wound usually suppurates. Adhesive plaster may be applied, in order that the divided parts may be brought into contact. In the course of twenty years' practice, I have invariably found that the after-treatment of the patient is not of less importance to his life than is the operation itself."

The reasons Mr. Carpue gives for preferring this operation, are,

"1. Because it is generally performed in less time than the lateral operation.

"2. There is less pain.

"3. There is no fear of a fatal hemorrhage.

"4. There is no division of the prostate, nor of the inferior part of the bladder; nor is there any danger of wounding the rectum.

"5. The stone, if of a certain time, cannot be extracted by the lateral, but may be extracted by this method.

"6. A small stone is more readily discovered by this method than by the lateral.

"7. If the stone breaks, the particles can be extracted with more certainty than in the lateral operation.

"8. If the stone is concealed in a cist, the cist can be destroyed and the stone extracted, as is proved in Sir Everard Home's case; and, if the stone should be situated above the prostate, or in any cavity which is occasionally found in the bladder, it can with greater ease be discovered and extracted. There is also no danger of including part of the bladder with the stone, in endeavouring to extract it, nor any danger of a fistulous opening after the operation.

"9. In case there should be any disease of the bladder, it can be examined, and proper means prescribed for the cure."

To these, another may be added, which we consider by no means of trifling importance; and we think, all those surgeons will agree with us that have seen much of *first operations*,—that it may be performed with more facility, and even dexterity, on a first occasion. Who has not seen young surgeons fail in their attempts to make the incision in the bladder; run the gorget out of the groove of the staff, perhaps into the rectum; make many different incisions into the bladder; thrust the gorget quite through it; grasp the bladder or the prostate gland in the forceps," &c. &c. These circumstances are descanted on by Mr. Carpue; and he shows that the occurrence of some of them cannot be always avoided by persons possessing the most accurate anatomical knowledge and greatest manual dexterity. The chief cause of several of these accidents consists in the varieties occurring in the situation of the bladder and its connexions. " If we consider the situation of the bladder and the parts connected with it," says Mr. Carpue, " (such as the levatores ani, the obturatores interni, the coccygeian muscles, the muscles from the rami of the ischium,) we shall find that these muscles act upon a part (*i. e.* the bladder), which is but slightly attached to the pubis by cellular substance, and by the lateral ligaments: these, I find by repeated dissections, are seldom the same in different subjects. I say, if we consider the action of these muscles, it will be evident that the situation of the bladder must vary under different circumstances:" and he proceeds to show the influence they will have on the steps of the operation, and to trace the numerous serious accidents that thence commonly ensue.*

---

* As the anatomy of the muscles situate about the bladder and rectum, is not generally understood with sufficient correctness, because the parts are not examined, as Winslow directs, *in situ*, or else it is forgotten by those who have long relinquished their studies of this kind; we feel confident that we shall present some useful information to many of our readers, in giving Winslow's demonstration of those parts: for the works of this great anatomist are not in the possession of every surgeon, and no other writer has described all their connexions with equal clearness and precision. These extracts will form a good commentary, too, on the passage in the text from the work of Mr. Carpue. In sect. viii. No. 659, of his Anatomical Exposition of the Human Body, Winslow says,

" On each side of the bottom of the pelvis in both sexes, opposite to the lower part of the bladder, there is an aponeurotic or tendinous ligament, which runs over the inner surface of the musculus obturator internus from before backwards. The anterior extremity of this ligament is fixed on one side of the middle portion of the symphyses of the ossa pubis; and the posterior extremity to the middle part of the ligamentum sacro sciaticum described in sect. ii.

" 660. A little above the elongation called the neck of the bladder, the forepart of which is narrow and fixed to the anterior extremity of the ligament already mentioned, and the broad posterior part to the side of the bladder. These two lateral expansions may be looked upon as proper ligaments of the bladder, by which it is connected to the inner side of both ossa pubis.

" 661. To the anterior portion of each of these ligaments of the bladder, is fixed a particular fasciculus of fleshy fibres, which run up obliquely on the foreside of the bladder, on which those of each side meeting together, form a kind of muscular intertexture, and unite with the most transverse fibres of the bladder.

" 662. These two muscular fasciculi form a part, and perhaps the principal part, of what is called the sphincter of the bladder; but, to have a true idea of them, they must be examined *in situ*, without destroying any of their natural connexions. When the bladder is removed out of its place, as is done in the common method of dissection, these fasciculi are cut, and thereby their direction being lost, they appear transverse, and are taken, by those who know no better, for portions of an orbicular sphincter.

" 663. In males, these two fasciculi are partly fixed in the prostates; but in females they are very broad, and appear sometimes to be double on each side, one plane lying above the other. They are to be looked upon as true muscles, fixed by small tendons on the sides of the symphyses of the ossa pubis.

" No. 575. *Prostatici.*—The superior prostatici are two thin planes fixed in the upper part of the inside of the small rami of the ossa pubis, from whence they are spread over and inserted in the prostates. Their insertions in the ossa pubis are on one side of the obturatores interni.

" 576. The prostatici inferiores are small transverse planes, each of which is fixed in the symphysis, between the ramis of the os pubis and os ischium, and from

The only cases, Mr. Carpue considers, in which the high operation ought not to be performed, are, 1°, on a corpulent subject; 2°, where there is schirrus, or other disease of the bladder, so that the sonde-de-dard cannot raise it above the pubis.

On the other hand, "in patients where the staff cannot be passed, in consequence of stricture or disease of the prostate, or where the calculus is of a certain magnitude, there is no choice of the mode of operation. Either the high operation must be performed, or the patient is doomed to linger out a life of wretchedness."

We think that no other arguments than the above statements of facts are necessary, in order to render the advantages of the high operation, in the generality of cases, over any other known one, clearly evident. Amongst the histories of the cases in which it has been used, only two or three instances of death can be imputed to the operation; and, indeed, there is only one of those above referred to, which would not have been, at least, equally likely to have happened from the lateral method. Now, Dr. Marcet says, in his Essay on Calculous Disorders, "there is reason to believe, as will be seen in the course of this work, that the average proportion of deaths from the operation, is no less than one in five;" and he adds, that "he has reason to believe that the number of fatal cases in Guy's Hospital, is as three to twenty; and that the number of deaths in La Charité, is one in five or six." The proportion of fatal cases in the Norfolk and Norwich Hospital, comprising the years from 1772 to 1816, has been four to twenty-nine; nearly all the surgeons of which, during that period, have been distinguished men.

We were nearly neglecting to transcribe Mr. Carpue's direction for performing the high operation in females: the following is the method of it.

---

thence runs transversely till it meets its fellow from the other side under the prostates, to which they are both strongly connected, and they serve like a girth to sustain these glands. They may be considered as two small or internal transversales; and the other two transversales may be distinguished by the names of great or external. They have likewise some adhesions to the point in which all these muscles hitherto described are united."

The description of the other most important muscle, we shall take from Fyfe; which is the "*levator ani. Origin.* By a semicircular edge, from the os pubis, within the pelvis, at the upper edge of the foramen thyroideum, from the aponeurosis which covers the obturator internus and coccygeus muscles, and from the spinous process of the os ischium; its fibres descend like rays from a circumference to meet those of its fellow, and with it to form a kind of inverted funnel.

"*Insertion.*—Into the sphincter ani, accelerator urinæ, and under and fore-part of the os coccygis. It surrounds the extremity of the rectum, neck of the bladder, prostate gland, and part of the vesiculæ seminales.

"*Action.*—To support the contents of the pelvis, to retract the end of the rectum after the evacuation of the fæces, to assist in the evacuation of the rectum and bladder, of the vesiculæ seminales, and prostate gland. It is likewise considered by some as a principal agent in the distention of the penis, by pressing upon its veins."

"The incision is to be made in the integuments.

"The sonde-de-dard is to be introduced by the meatus urinarius into the bladder.

"The incision into the linea alba as in males.

"The stilet is to be passed through the upper part of the bladder; the probe-pointed bistouri is to be introduced into the groove of the stilet, and an opening made into the superior part of the bladder; the stilet is to be withdrawn. The index-finger of the left hand is to be introduced into the bladder, to suspend it. A suspensor of the bladder is now to be introduced, which is to be held by an assistant, and the stone is to be extracted. A flexible gum catheter is to be passed into the meatus urinarius, and retained in the bladder."

We shall close our analysis of this interesting work with the following account of what has transpired respecting the high operation in England, since Mr. Carpue, from having witnessed it at Paris, (by Mr. Souberbielle, who has performed between twenty and thirty successful operations on male subjects, all from fifty to eighty-six years of age, except one, who was fifteen,) has endeavoured to introduce the use of it here, according to the method of Frère Côme.

"On my return from Paris, in 1818," says Mr. Carpue, "I waited upon Sir Joseph Banks, (whose anxiety to be informed of, and to encourage, whatever may be for the benefit of society, in any branch of science, is so generally known,) in order to exhibit to him the apparatus for the high operation, and the cast of a large stone which had been extracted with success by Dr. Souberbielle. At Sir Joseph's, I had the pleasure to meet Sir Everard Home, whose pupil I had the honour, some twenty years since, to be. On looking at the calculus, Sir Everard said, that "it could not have been extracted by the lateral operation." On the following day I received a letter from Sir Everard, desiring me to show him the instruments that were used in the high operation, and to inform him of every particular connected with the important subject of its performance. With these requests I cheerfully complied. All who have the advantage of Sir Everard's acquaintance, are well aware that, though quick of apprehension, he never ventures an opinion without being fully master of the subject; nor was he at all satisfied with the propriety of the high operation, till I informed him of the opening in the perinæum. A short time after this I received a second letter from Sir Everard, saying, that he was so well satisfied of the advantages of the high operation, that he had a patient on whom he intended to perform it; but at the same time politely added, that, if I had an operation in view, he would defer his till mine had taken place. Being anxious to have the operation performed in this country, and more particularly by the hands of Sir Everard Home, I sent him the instruments which I had brought from Paris, and accompanied him to St. George's Hospital on the day of the operation.

"The operation was somewhat lengthened, in consequence of the stone being in a cist; yet its duration was very short, in comparison with what it would have been, had it been performed in perinæo. I am enabled to state the exact time, as a friend, who held a watch in his hand, stated to me that the operation lasted exactly seventeen minutes. In this case the stone was with difficulty felt by the sound; and, when Sir Everard introduced his finger into the bladder, he could not feel it: however, he soon discovered the cist, which he was compelled to break, and then readily extracted the stone. I received the stone from him, and observed part of the cist adhering to it: this I detached with my nail. The boy recovered, and is at this time in perfect health."

Although we have chiefly confined ourselves to those points tending directly or indirectly to show the advantages of the high operation, yet we have, we think, given the reader some idea of the merit of the contents of the present work. Besides the many valuable critical remarks on the different methods, he will have perceived that it presents the first accurate history of lithotomy that has been written; for, in addition to several other important omissions, no former writer had shown the claims of Franco to the invention of the three operations,—the *lateral*, the *high*, and that *en deux temps*. The last has been passed over unnoticed, and the first generally attributed to Frère Jacques. As the works of Franco are extremely scarce, (we believe there is but one copy in England,) Mr. Carpue has given, in the original, that part of them relating to the subjects under consideration: he has also inserted in his work *fac similes* of the plates in that of Franco, describing the instruments employed in the operations in question by this great improver of the art of surgery.

---

*A Sketch (analytical) of the History and Cure of Contagious Fever.* By Robert Jackson, M.D. 8vo. pp. 284. Burgess and Hill, London. 1819.

Fever, in some form or other, the most common as well as most important of the diseases to which human beings are liable, engaged the attention of Dr. Jackson in a particular manner on his commencing practical observation, and he has never ceased to pursue the investigation of this subject during a period of nearly thirty years, spent in active duty in the British armies in almost every quarter of the globe. In 1817, Dr. Jackson published the results of his observations and reflections on the nature and cure of endemic fever; and, in order to illustrate still further this part of pathology, he now adds a similar history of fever which is contagious.

Contagious fever, as it appears in fleets and armies, is, according to the opinion of Dr. Jackson, almost, if not always, an artificial disease; that is, the product of the accumulation of many persons under canvas in the field; in the confined space between decks of ships, or in damp and ill-ventilated barracks. It has often a similar origin among masses of manufacturers, shut up in ill-ventilated workhouses, or lodged in damp and ill-ventilated rooms, as places of dwelling. The disease, as originating from a common cause, is analogous to general fevers; it is modified more or less in appearance by circumstances of place and subject. A confined atmosphere, under the circumstances above designated, acts adversely on human health. It occasions a disease, which, in the course of its progress, generates a material which is communicable to others, and which, thus communicated, propagates its kind through a series,—in other words, becomes contagious.

This is the source of contagious fever: but fever sometimes appears suddenly, and extends rapidly and widely over an extensive country. Its rise, at least its progress, cannot be supposed to depend on a state of atmosphere locally corrupted by artificial aggregation; it must be supposed to depend on something adventitious connected with the constitution of the atmosphere generally diffused in it; and thus diffused and predominant, is distinguished by the term epidemic. Different portions of the annual revolution, as marked by different conditions in the state of the atmosphere, are for the most part distinguished by peculiarities in the form and feature of the diseases which affect the human race. This is common and regular; and, as connected with the operation of sensible causes, is supposed to be comprehensible, as far as human powers are capable of comprehending the causes of things. But, besides what is now stated, certain new and inexplicable effects arise occasionally, after the lapse of several annual revolutions, so irregular in time and mode, as not to be calculable, but so impressively marked in their steps, as to command attention. The duration of epidemic influence is uncertain: sometimes six weeks; sometimes two months; sometimes six; and sometimes for a series of years, with occasional risings and fallings, or cessations and recurrences, within the extreme limit. During their prevalence, diseases usually present themselves with peculiarities in character, and spread or propagate with a facility unknown at other times.

Febrile diseases have many forms, and almost an infinity of shades of form. They are sometimes epidemic without being contagious, or susceptible of contagion; sometimes they are epidemic, and though not originally so, are readily convertible to contagious action; or rather, are with difficulty prevented from assuming it. Of these, the gastric form of fever is the most prominent; and it is the form which most prevails in Great Britain, and has been predominant for the last three or four years.

Sometimes the local action developed in fever connected with a contagious cause, is developed in other parts, as the intestines, (appearing as dysentery,) the lungs, and the brain. And a pustular affection of the skin, terminating in ulcer, apparently from the same cause, is not rare.

The means which effect the change in the action of parts above alluded to, must be necessarily considered to be the means of an irritative nature; inasmuch as they produce a new form of life, as a result of excitation. The noxious cause, which floats in the atmosphere, and which is applied by its means to the surfaces of the skin, interior (the mucous membranes), and exterior, after a time, which varies from twelve hours to several days, or some weeks, irritates the parts to which it is applied, and produces an action which generates a material similar in kind to that by which it was itself created.

When the local affection from contagious matter is carried to a certain point, it propagates its kind through the mechanism of animal organization. The proper act of the contagious cause is necessarily supposed to move in one series of parts only; the act more or less intense in degree, and subject to variety of complication from accidental contingencies. Those parts are the secretory vessels or organs. But, though the generative act of contagious fever, considered as a generative process, moves on one series of parts only, and after one mode of proceeding, yet, as the animal body is a complicated machine, connected in all its points, the whole of the system is more or less implicated in the process which is necessary to the generation of the contagious material. When fever presents itself under a complicated form, the symptoms which are mostly contiguous, independent of the process of contagion, are often so varying and fluctuating in their course, that it is not easy to analyse the history correctly. Besides those of nearly all the abdominal organs, the functions of the lungs, the heart, and the arteries generally, are much disturbed. The intellectual organ, and the nervous system universally, are variously affected. The state of the skin is always more or less changed from its natural condition; changed in such a manner, in most cases, as to furnish a diagnostic of the disease.

Fever, then, may be said to be the act of the general system: but, in saying this, it is not understood that every part of the system is equally under the febrile act. It is reasonable to believe, that the impression of the febrile cause is made upon a surface, or a point of a susceptible surface, presumptively the cutaneous expansion, exterior or interior. The impression is conveyed, through channels not distinctly known, to the common centre of the system, the seat of life and motion; and thence, if one may use the expression, it is dispatched to the series upon which it was originally made, with a power productive of effect; that is, generative of formal disease. The impression is made upon an exterior part or extremity; it receives force, and becomes fever through the instrumentality of the sensorium; the sensorium is not the seat of the disease; and it may be added, that, where the substance of the brain, or its membranes, is affected, the effect is contingent and secondary, as effects on other analogous structures. The whole series of the structure, upon which the impression is made, participates in the diseased act; the act is, however, often expressed more prominently in one part of it than on another: a prominence arbitrarily and contingently changed in the course of the duration of the disease. The impression is made primarily on one series; the effect is occasionally extended to others; it may exist in more than one; its activity is prominent only on one at one time.

The most important principles of the above doctrine have already been marked by our approbation, on several occasions, when the opinions of M. Broussais have been the subject of consideration; we need not, therefore, now dwell on them: but there are two points about which this physician and Dr. Jackson differ. M. Broussais is conscious that the first impression giving rise to the phenomena of fever always affects the mucous membrane of the stomach, either from the exciting cause having been immediately applied to this organ, or from its sympathising with some remote part in a state of irritation; and, unless such a sympathy happens in the latter case, he considers that real fever cannot arise. Dr. Jackson equally inculcates the doctrine of the local origin of contagious fever, but he does not fix this origin in any part exclusively; and he, besides, considers that the skin itself, properly speaking, or the cutaneous envelopment of the exterior of the body, as well as the mucous membrane of the lungs and alimentary canal, may be the part first affected, and that the irritation of this organ may excite in the system the whole of the phenomena to which the generic term *fever* has been applied, without the mucous membrane of the stomach being necessarily interested in the production of the series of symptoms: though he states, that such an interest does take place in by far the greater proportion of instances. On these points we agree with Dr. Jackson.

The other point of difference, is the part of the nervous system which forms the medium through which the symptoms are developed: though the reality of a difference in this respect is not very evident. Dr. Jackson designates it as the *sensorium;* by which it appears he means the cerebral system: though he may intend only to signify the *principle of sensibility* generally, without specifying the particular parts in which it may be seated. M. Broussais considers the ganglionic nerves as the essential organs by which the sympathies in question are produced; and many powerful arguments may be adduced to show the propriety of this notion. The secondary and more remote affections can often be traced, (in the more simple forms of fever, and when the progress of the symptoms is slow and gradual,) developing themselves according to the order and relations of the functions of organic life in the state of health; and the functions of the cerebral system of nerves frequently continue undisturbed, though all the phenomena of fever be present; but this is never the case with respect to the ganglionic system, and the functions dependant on these are always deranged in a direct ratio of the degree of the fever: or, as it would be more proper to say, the violence of the fever is in a direct ratio of the derangement of the functions of the ganglionic system.

The questions above stated are really worthy of the most rigorous examination; but we shall turn from them, to what we believe will be more interesting to the greater part of our readers, —that part of the work of Dr. Jackson which relates to the cure of fever.

Dr. Jackson's mode of cure of fever arising from contagion, is a result of his theory; and the advantages of it have been proved by many years' experience. It was his theoretical views which led him, above twenty years since, to use blood-letting as a common measure in the treatment of that malady: a period when such a practice was generally reprobated as murder, direct or indirect. But, in the revolution that has since taken place in medical doctrines, and which has exerted an impressive influence even on empirical practitioners, blood-letting has become not only admitted amongst the remedies, but extolled above all other curative means. But there is one point in the therapeutics of Dr. Jackson which is in a manner still peculiar to himself: this, too, has arisen from his theoretical speculations. We express ourselves in this way, because there are notions echoed about amongst medical practitioners of a certain disposition of mind, that practice will lead to improvements in the mode of curing diseases without reasoning on their nature; and that the latter is, indeed, really useless in this respect. With so forcible a refutation of at least one of those notions before us, it is hardly possible to avoid some remarks on them, although the subject has long been a common-place theme.

"It is a fact which no one will dispute, (says the author,) that diseases are cured by subversion of the diseased action at its proper base: it may therefore be presumed, that it (contagious fever) is most speedily and most effectually cured by applications which act directly and immediately upon the series of parts affected." This indication, and his mode of fulfilling it, are points on which the author rests his principal claims of improvement in this part of the practice of medicine.

"If a change in the mode and balance of action in the *organic series of the animal system*,"* says Dr. Jackson, "be admitted to be essential to the existence of fever, the arrest of the diseased course by such means as are best calculated to effect the arrest, with the least chance of endangering general life, is necessarily the first step in the medical proceeding. When the arrest is effected, the reproduction of the movements of life to their customary order and activity, by suitable stimulation, is the next; and it is mainly important. The proceeding is thus of a double character: its act is force throughout; not force against nature, but against the action of the cause which subverts the order of nature. The mode of execution varies according to circumstances, viz. quantity of momentum in the diseased act; nature and constitution of the part upon which the act is manifested; condition and habit of the individual contingent at the time, or constitutional. The cause of endemic fever seems to be directed to parts of comparatively gross structure; the product is visible and gross; the means of remedy strong, as corresponding with the condition of the disease. The cause of contagious fever, in so far as respects the contagious act, is directed to parts of extreme subtility, presumptively the organ of invisible exhalation, which is diffused through the whole of the cutaneous expansion, exterior and interior. The cutaneous expansions seem thus to be the direct seat of the disease; and, in correspondence with this opinion, remedies, in order to be effectual, are directed to be applied to these expansions in various ways and manners. The cure of contagious fever may thus be said to be laid on a basis of theory; experience proves decidedly that, so laid, it is laid upon a basis of fact."

We shall pass in review the particular measures which the author adduces as proper to fulfil the above indication. Blood-letting he has been accustomed to resort to since the year 1796, "without fear of doing harm, generally with benefit; at least, with such benefit as to render the disease tractable to other remedies." Emetics are of great value: judiciously managed, they often cut off the disease in its beginnings. He prefers those "of severe operation, such as occasion sickness of long duration." Purgatives of brisk operation, namely, jalap with calomel, emetic tartar or James's powder, and a small quantity of opium, have singular good effects in diminishing violence and danger, where the symptoms indicate mesenteric congestions, or where the disease is accompanied with torpor or irregular action of the bowels. Blisters applied to the temples, nape of the neck, and extended down the spine to the interval

* By this expression, it would appear that Dr. Jackson and M. Broussais agree in the second point of doctrine above particularly designated; but he has no where else expressed himself in this manner.

between the shoulders, were frequently employed, even, he states, at the *earliest stages* of the disease, with a marked good effect. Warm fomentations to the feet and legs, scrubbing the skin with soap and brushes, warm bathing, followed by cold affusion, &c. often arrested the disease. Gestation in the open air, continued for a length of time, was a prescription of necessity, oftener than design; but it was mainly conducive, when employed, in confirming the health that was restored by the judicious and prompt application of the means now mentioned. The contagious action of the disease, Dr. Jackson thinks, may be extinguished by the prompt and vigorous application of the means above stated; or at least, that the disease will be put into such a train, that a favorable termination may be expected at the seventh day, or first critical period.

Here it is necessary to state, that the author's observations have led him to believe confidently, that the morbid actions in contagious fever proceed in certain trains, occupying determined periods, and when they recur, that they again run through an analogous course; though he has not always remarked any particular evacuation at either of the *crises* here designated.

The more simple form of fever from contagion, usually runs on progressively, with risings and fallings until the fifth, oftener until the seventh, day, when the skin, which had been hot and dry, begins to become moist, and sometimes covered by fluid perspiration. The urine often deposits a sediment, and the organs in general resume their healthy functions. The absence of the fever, thus attained, is of various duration; sometimes of three days or more, sometimes only of a few hours. The symptoms recur; and, thus recurring, proceed on the same base. About the fourteenth day from the commencement of the disease, the seventh from the recurrence, the course terminates in crisis, or the mode changes to another form. The termination is sometimes final; sometimes it is succeeded, at a short, or at a scarcely perceptible, interval by another series of febrile symptoms, which carries the disease on to the twenty-first day.

This form of fever, in by far the greater proportion of cases, terminates favorably under the use of the measures above designated; but there is a form of fever which is more complicated with respect to the organs especially affected, and which does not move on in the regular progressive manner of that just described.

"It sometimes presents itself with appearances of violent, irregular vascular excitement, and local determinations which threaten convulsion and apoplexy, suffocation or engorgement of internal organs, lungs, liver, or spleen; sometimes with a strong, or what may be called a concentrated, general action, thickened and constricted skin, ardent as a live coal; a condition, which threatens to subside by internal congestion, or to explode by local external gangrene of varied form, viz. petechiæ, streaks of ecchymosis, or extensive and deep blacknesses. The basis of the cure is common; the means are similar as in the preceding; the application is modified by circumstances. If a person, labouring under this form of the disease in either of its conditions, be submitted to treatment within twenty-four hours from the time of the attack, abstraction of blood is evidently the first step in the physician's course. When a certain quantity of blood, only measureable by the physician as superintending the process, has been abstracted from the arm, it is advisable that the patient be stripped naked and immersed in a warm bath of moderate temperature, the immersion continued for fifteen or twenty minutes, the skin strongly scrubbed with brushes and soap while under immersion. At the expiration of twenty minutes, the condition is to be examined with care; and, if it be then found that the mode of action has not changed, that is, if the movement has not become general and equal, it will be advisable to re-open the vein, and to allow blood to flow, while the patient remains in the bath, until the object in view be attained: that is, until the circulation be in some manner equalized. When that is done, cold water is to be affused copiously upon the head and shoulders, while the lower extremities remain in the warm bath. The course of the disease will, in most cases, be suspended, if not perfectly arrested, by the effect of the proceedings now recommended; and when that is done, the body, being removed from the bath, is to be wiped dry, and laid in bed; blisters, as means preventive of return, are to be applied to the head, neck, back, or sides, according to the predominance of the local symptoms. Friction with warm oil will be useful; emetics and purgatives promise the same benefit here, after the case is simplified, as in the preceding. A bolus of camphor, nitre, tartarized antimony and snake-root, with half a grain of opium, and two or three grains of calomel, given every five or six hours, with plentiful dilution, frequent ablution, and frequent change of bed and body linen, conduce, if the course of the disease be not totally arrested, to maintain the movements in an equal tenor until the febrile circle be completed, when healthy action may be expected to re-appear. The outline of practice now suggested, varied according to circumstances, applies to the cure of the disease in all its conditions, whether as preternaturally excited, or as constricted through excess of force."

In cases of relapse in the more mild and simple form of fever, but little medicinal aid was required.

"Attentive nursing, diluting liquids, pure air, frequent change of linen, occasional fomentations of the extremities with flannels wrung out of hot water, ablution of the body with warm or cold water, according to the circumstances of the case, are, for the most part, sufficient to maintain the course in progress until signs of termination manifest themselves: that generally happens at a noted critical period. But if, instead of an open course of regular progression, symptoms occur which threaten convulsion or other violence, the patient is to be immersed in a warm bath, a vein opened in the arm and blood abstracted, while he is immersed, in quantity sufficient to make impression on the action then existing. The head is moreover to be shaved, covered with cloths wet with vinegar and salt, blisters applied to the back part of it, to the spine, continued from the nape of the neck to the interval between the shoulders. Camphor, nitre, James's powder, with calomel and opium in bolus, is often given with advantage in this condition; it is here that cobweb appears particularly serviceable, more serviceable than opium.

"Another condition, connected with contagious fever in its secondary course, is characterized by a small and frequent pulse, muttering or faultering speech, confused intellect, extreme weakness or inability. It is essential to ascertain, in this case, in so far as is possible, whether the existing symptoms are the sequel of highly-excited action, or if they are connected with contingent local oppression on the organ of sense and motion. In the first, it is almost always proper to cut off the hair, to shave the head, perhaps to apply blisters to the scalp and other parts of the body, to give internal cordials to the extent of impressing the system perceptibly, not of exciting action inordinately. Camphor, snake-root, with more or less of the acetated water of ammonia, or James's powder; calomel in small doses; fomentations of the extremities with flannels wrung out of hot water; immersion in the warm bath; and, after removal from the bath, friction of the body with warm oil, volatile liniment, &c. aspersion of the surface with cold salt water, with *eau de cologne;* frequent change of bed and body linen; pure air, warm apartments, gestation in the open air in a convenient vehicles comprise the principal of the curative means: the application to be varied in mode and degree, according to the circumstances of the case. In the second, evacuation is necessary, even abstraction of blood by cupping, or by the lancet; the operation to be performed while the body is immersed in a warm bath. Blisters are to be applied to every open surface about the head and upper part of the spine. Calomel, with some grains of James's powder, and a proper quantity of powder of jalap, is to be given as a purgative, followed by plentiful dilution, long-continued fomentation of the extremities with flannels wrung out of hot water; in short, by evacuation combined with stimulation in such a manner that, while oppression is relieved by the one, recurrence is prevented by the other, as an effect of counteraction."

Sometimes the diseased action is so prominently manifested in some particular organ, as to obtain a name of local distinction; and many cases of this kind require some modification of the means that are ordinarily applied for the cure of the general disease. When the surface of the alimentary canal is thus affected, especially that of the stomach, the exhibition of an emetic, at an early stage, may be considered the first remedy, with respect to time. "Where the emetic acts effectively, or rather severely, the result is often decisive of cure, particularly if followed by a brisk purgative, namely, calomel with jalap and James's powder, or emetic tartar, ablution with cold water, and exercise in the open air." Where the tongue is preternaturally red at the commencement of the disease, whether rough, or smooth and shining, emetics are less proper, or less effectual. Here the measures usually termed expressly antiphlogistic, must be chiefly depended on.

In the dysenteric form of contagious fever which occurs sometimes as a primary affection, especially in damp and foggy weather, in camp or bad quarters; frequently as a secondary malady, in crowded and damp military hospitals; the first object, in the cure, is to produce an equalization of the circulation by immersion in a warm bath of rather high temperature; by abstraction of blood, either by the lancet, by cupping, or leeches; by vesicatories applied over the whole abdomen; by emetics of ipecacuanha, followed by purgatives; namely, calomel, rhubarb and charcoal, with a certain portion of James's powder, or emetic tartar, and sometimes a small portion of opium. These means, judiciously modified according to particular circumstances, but rarely failed in arresting the diseased action, or in giving the action such a tendency as led to a favorable issue. In the more advanced stages, or in the condition termed chronic, the means of remedy vary exceedingly. But, the author adds, "many physicians rarely have it in their power to apply what is proper, either in diet or regimen; and hence the mortality in the dysenteric columns of military-hospital returns is almost always enormously high."

When the lungs or brain are the organs especially affected, more copious blood-letting, with the other measures designated for the cure of the second form of fever, should be more freely resorted to.

The management of patients during convalescence does not here differ essentially from that proper for endemic fever, which the author treated of in his work on this subject: but there are some remarks on the influence of heat, produced by fire, in the wards of fever-hospitals, that we think too interesting not to be added to the view we have given of this part of the work.

"Ventilation, or the ingress and egress of the common atmosphere, will be sufficiently secured by the means proposed; and, in warm and dry weather, ventilation will be sufficient security against the accumulation of contagion. In cold, damp, and foggy weather, the free admission of external air might probably be injurious by its impression: air of that description is moreover not calculated to absorb or dissipate the floating contagion. In this case, the strong heat of fire in open fire-stoves, so placed as to diffuse its influence into the lower layer of the atmosphere, has appeared to myself to be the only substitute for defect of common ventilation, and the only sure means of rectifying the air that is vitiated by emanation from the bodies of living men. Heat, as acting on the skin, probably operates favourably on the conditions of contagious fever; it evidently operates favourably, as exciting and maintaining circulation of air within the apartment. I

here take leave to mention the circumstance that first directed my attention to it. The wards in the barrack in Westmoreland Fort, on Spike Island, which were allotted to the reception of the sick of the St. Domingo expedition, and which were crowded to the most extreme degree of crowding, were also in some degree cooking-places; that is, employed for the preparation of the lighter parts of diet. A large fire was necessary; and a long grate, being filled with coals, threw out a great heat, sufficient to roast a surloin of beef. This was the case in the larger wards, where there were from forty to fifty persons stowed on the floor as close as they could lie. Those who lay within a certain distance of the fire, generally did well, though the symptoms of the disease were often violent; those who were near the end where there was no fire, died in great numbers, though the symptoms of the disease often appeared to be moderate. The air near the fire was comparatively light, and not offensive; near the remote end, it was heavy, unpleasant, almost insupportable to the transient visitors. The influence of strong heat from fire, in a sick apartment, appeared to be so useful, at least so agreeable, in the present case, that large fires were ordered to be made in all the huts and sheds on the outside of the fort which were occupied by the sick. The air in these huts, though filled with sick to overflowing, was not offensive, the progress of contagion was not active, and mortality was comparatively moderate; in fact, on the lowest scale. From that, and other more recent experience, I am disposed to consider the action of the heat of fire as a most important mean of ventilation, mainly conducive in arresting the progress of contagion; the chief trust, in fact, in damp, foggy, and still weather, through which we can expect to preserve the air of hospitals, as filled with febrile sick, from a dangerous vitiation."

Here we conclude our account of this work. We have given a comprehensive view of the author's pathology, and the most important of his general principles for the treatment of contagious fevers: but, there is one part of this treatise, not inferior in value to that we have noticed, that we have wholly neglected; this is, what relates to military medical police. To give an abstract of this, so confined as the limits of our Journal must require it to be effected, would be to do great injustice to the author. The results of thirty years' observation, during active official duties under a multitude of varieties of climate and other topical circumstances, cannot be thus pourtrayed in their true character. It is for the same reasons that we have not passed the different sections of this treatise in review; but have chosen to make at once general abstracts from such parts of the whole, as we have considered best calculated to give some idea of the character of the work. This, then, is probably the last production of the pen of Dr. Jackson: it completes the history of the subjects, to the illustration of which the labours of his life have been chiefly directed; and we think no one will differ from us in the opinion, that the results of them must be placed amongst the most valuable productions in medical literature.

*A History of the Introduction and Use of Scutellaria Lateriflora (Scull-cap), as a Remedy for preventing and curing Hydrophobia, occasioned by the bite of Rabid Animals; with Cases, accompanied with a Plate of the Plant.* By LYMAN SPALDING, M.D. Read before the New-York Historical Society, September 14, 1819. 8vo. pp. 301. Collins and Janson, New York; and Souter, London. 1819.

THERE is something in the moral conduct of the generality of us, we know not precisely with what terms to qualify it, whether as professional dignity, pride, or prejudice; but there is some feeling of this kind which undoubtedly deprives us of much valuable medical knowledge. Several of the most valuable remedies and medical measures we are acquainted with, were in use amongst the people for a long period, perhaps several centuries, before physicians deigned to make enquiries respecting their merits; and, in many instances, those who, deviating from the common course, have resorted to such measures, have acquired a degree of honourable fame, that nations are never reserved in conferring on their real benefactors.

The use of scullcap as a preventive of hydrophobia, appears to have been resorted to for half a century in some parts of the United States of America. The first person, as far as Dr. Spalding has been able to learn, who employed it, was Dr. LAWRENCE VAN DERVEER, of Roysfield, in New Jersey. He is known to have done this in 1773, and to have continued it until the time of his death, which happened in 1815; but it cannot now be ascertained from whom he obtained the knowledge of its antidotal powers.

"It has been suggested," Dr. Spalding continues, "as probable, that he became acquainted with the virtues of this plant, during his residence in Virginia; but this cannot be correct, since he was known to have used it in New Jersey, before he removed thither; besides, on a reference to the many nostrums which have been celebrated for preventing hydrophobia, we do not find that scutellaria had been used either in Virginia, or in any other place, previous to its employment by the doctor. Our inquiries do not lead us to believe that the doctor kept his remedy a profound secret, although he had been accused of it by many; but so much do medical men despise what they consider vulgar specifics, and so little faith do the public place in them, that this remedy for forty years was scarcely known or heard of beyond the doctor's immediate circle of practice. It was from these circumstances that no one had the curiosity to ask this gentleman how *he* came by a knowledge of the antidotal powers of scutellaria. From the upright unassuming character of Dr. Van Derveer, his correct moral deportment and regular medical standing, we are led to believe that he would as frankly have communicated the source of his information, as the remedies used.

"Among the many persons to whom he communicated a knowledge of his remedies, may be numbered Drs. Morris, Kinney, Little, Henry, and Bloomfield, of the revolutionary army; Dr. Henry Schenk, sen. Daniel, Lewis, and Dr. Henry Van Derveer."

Dr. Van Derveer does not appear to have kept notes of the cases in which he employed this remedy; but, it is asserted that he had administered it to about four hundred persons; in none of whom, excepting the following, did any symptoms of hydrophobia appear. This was a man who, with another, had been bitten by a rabid cat; "they both applied to Dr. Van Derveer, and he gave them the scullcap. One of them took it, according to his directions, and suffered nothing from the bite; the other, by accident, lost almost the whole of his infusion, and could not be prevailed on to procure any more; because, from the smallness of the wound, and other circumstances, he supposed there was no danger. On the 23d day after the bite, this man was brought to Dr. Van Derveer in the last stage of hydrophobia, and died in eight hours after his arrival."

One instance is related by his son, of apparently well-marked hydrophobia, occurring after the bite of a dog who died rabid, being perfectly removed by the use of this remedy. He made, it appears, more than an hundred experiments on the antidotal powers of the scullcap; in each of which the remedy was given to a part of the bitten animals none of these were afflicted with hydrophobia; but, in every instance, some of the animals, which did not take the scullcap, died rabid. Many analogous results from its use are adduced by Dr. Spalding, on the testimony of physicians of respectability.

A man of the name of Lewis, of West-Chester county, New-York, was bitten by a mad dog in 1783, and went to Dr. Van Derveer, who gave him directions for the use of this remedy; and this man continued to administer it to his neighbours and their animals with success, until his death, in 1810. The number of cases to which he applied it, are stated by the people to have been one hundred persons, and twenty or thirty animals.

Numerous cases, testified by persons apparently worthy of credit, and apparently proper for putting its powers to the test, are related, in which the family of this Lewis had also employed this remedy with universal success. We shall give the following statements in detail:

"About five years ago," says Mr. Coleman, (Evening Post of 23d May, 1811,) "a mad dog, at Pelham's manor, being a small favourite, and running about the house, bit no less than nine persons in one family: to these the scullcap was administered by Lewis without delay, and no one experienced the least inconvenience. Two hogs in the neighbourhood were bitten also by the same dog, at the same time, which were shut up, and nothing was administered; both of them went mad, and died of hydrophobia."

"In the Evening Post of the 21st December, 1811, Mr. Coleman says, 'I have myself seen a convincing proof of the efficacy of Lewis's remedy. A couple of hogs in the neighbourhood in which I reside were bitten by a mad dog, and several cattle were also bitten by the same dog, some before and some after the biting of the hogs. Mr. Lewis was sent for, and gave his preparation to the two hogs, both of which, though much wounded, recovered without any signs of madness; while every one of the cattle having taken nothing to prevent the consequences, died of hydrophobia.'

"Mr. Albert Smith, student in medicine of New-Rochelle, has communicated to me the following cases:

"Some years ago, a son of Elijah Williams, esq. of West-Chester, and a black man belonging to the family of Isaac Briggs, were bitten by a mad dog. Lewis was employed to administer the scutellaria to them, and neither of them had any symptoms of disease; although three hogs and a sheep, which were bitten at the same time, died rabid.

"The Rev. Mr. Barlow has published the case of James P. Huntington, of New-Rochelle, who was bitten in the hand by a mad dog, in April 1811. He was treated by Lewis, and suffered nothing. Two of Huntington's blood-hounds were bitten at the same time: one of them ran away, and the other died with symptoms of rabies canina."

Several cases are related in which the remedy appeared to remove hydrophobia after its appearance; and one instance of this kind is given in detail by Dr. Spalding, in which the rabid state of the dog by whom the patient, a man, was bitten, and the existence of hydrophobia in the latter, appear well substantiated; in which the patient was attended by Drs. Stilwell and Robson; and a similar case, testified by Dr. Fisk.

The following are Dr. Spalding's conclusions:

"We have, then, the foregoing testimony that the scutellaria has been used by more than eight hundred and fifty persons, bitten by animals believed to be rabid; and in only three instances have symptoms supposed to be hydrophobic supervened, and in each of these cases the quantity of the plant actually taken was very inconsiderable. In two of them, the symptoms disappeared on taking more freely of the medicine.

"Furthermore, the scutellaria is said to have been administered to more than eleven hundred brutes, bitten by animals supposed to be rabid; and in no instance has any symptoms of madness appeared, excepting in the cases communicated by Dr. Bartlett.

"In more than one hundred instances, it is said that experiments have been made to test the antidotal powers of this plant, by giving it only to a part of the animals bitten; and it is stated that, in every experiment, those animals which did not take the scutellaria, have died rabid; but in no instance have any of those which took it had any indisposition."

