# Corrigenda in the Last Number

**Published:** 1820-04

**Authors:** 


CORRIGENDA IN THE LAST NUMBER.

Page 213, line 23, for *peritonæum* read *perinæum*; same page, line 37, for *peritonæum* read *perinæum*.

